# Supplementary figures and images for: Healthcare professional and commissioners’ perspectives on the factors facilitating and hindering the implementation of digital tools for self-management of long-term conditions within UK healthcare pathways
Source: PLoS One. 2024 Aug 23;19(8):e0307493. doi: 10.1371/journal.pone.0307493 (PMC11343405; doi:10.1371/journal.pone.0307493)

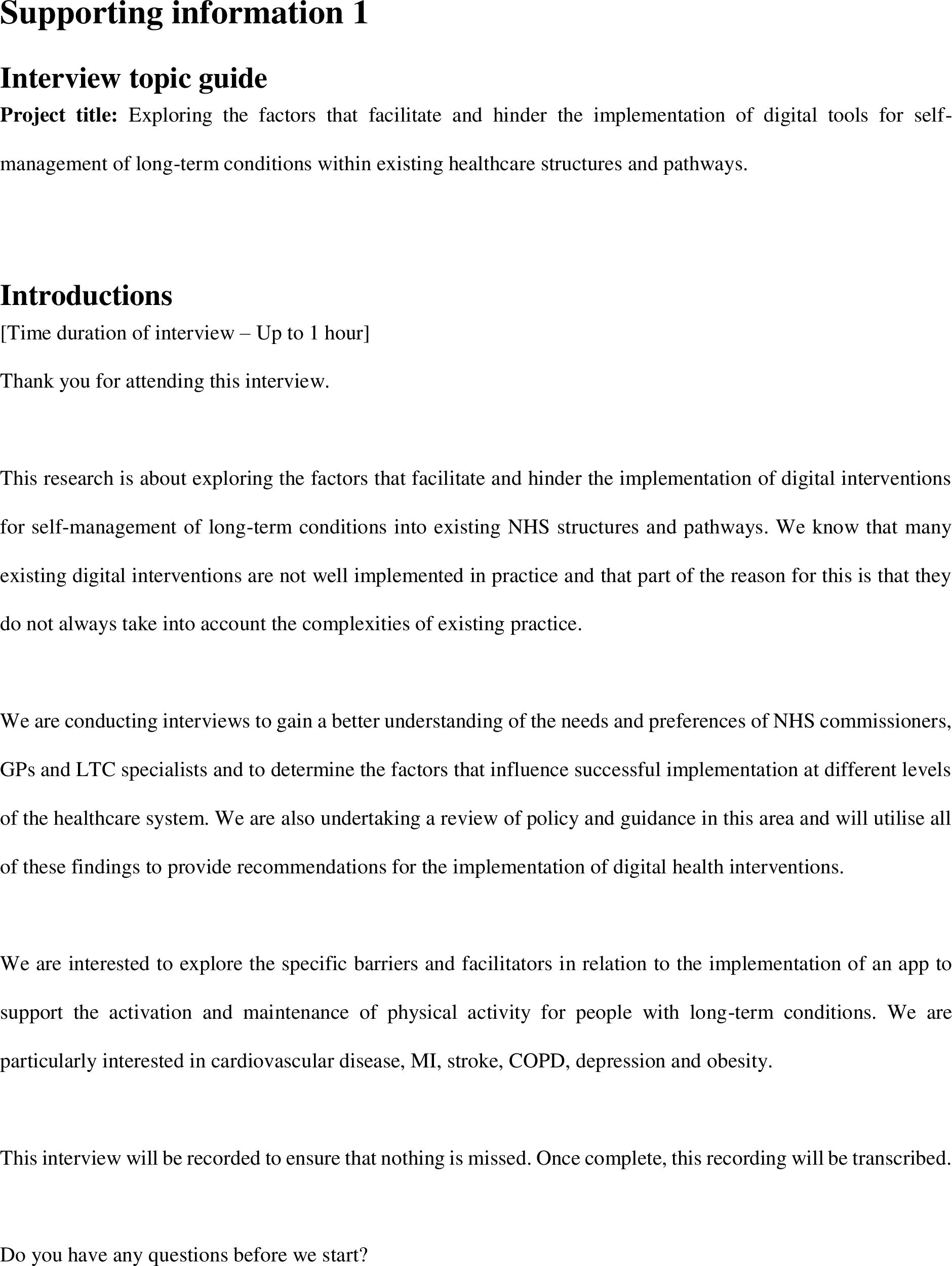

Supplement: S1 Fig — (TIF) [file pone.0307493.s001.tif]
