# Supplementary material for: Healthcare professional and commissioners’ perspectives on the factors facilitating and hindering the implementation of digital tools for self-management of long-term conditions within UK healthcare pathways
Source: PLoS One. 2024 Aug 23;19(8):e0307493. doi: 10.1371/journal.pone.0307493 (PMC11343405; doi:10.1371/journal.pone.0307493)
Supplement: Attachment — Submitted filename: Response to Reviewers [PONE-D-23-37603].docx [file pone.0307493.s002.docx]

**Response to Reviewers [PONE-D-23-37603]**

**Journal requirements:**

1. When submitting your revision, we need you to address these additional requirements.

Please ensure that your manuscript meets PLOS ONE's style requirements, including those for file naming. The PLOS ONE style templates can be found at

<https://journals.plos.org/plosone/s/file?id=wjVg/PLOSOne_formatting_sample_main_body.pdf> and

<https://journals.plos.org/plosone/s/file?id=ba62/PLOSOne_formatting_sample_title_authors_affiliations.pdf>.

We thank the editor for raising this. We have now revised the manuscript to adhere to the journal’s style, in particular using bold type for figure and table headings, adding the state/county for each author affiliation, and we have removed funding information from the Acknowledgments.

We have not added symbols for author contributorship, as it is difficult to ascertain individual, but equal contributions. Please instead see the Author contributions subsection (page 27).

2. Thank you for stating the following in the Acknowledgments Section of your manuscript:

*“We would like to thank members of the wider MOTH team and the National Institute for Health Research (NIHR) Applied Research Collaboration (ARC) Wessex staff, for their helpful involvement in the programme and administrative support, particularly during the COVID-19 pandemic.”*

We note that you have provided funding information that is not currently declared in your Funding Statement. However, funding information should not appear in the Acknowledgments section or other areas of your manuscript. We will only publish funding information present in the Funding Statement section of the online submission form.

We understand what should be included within each section, however, the Acknowledgements section quoted above, does not actually state any funding-related information. It is correct that the NIHR ARC Wessex were the funder, but their individual staff have also contributed to the work since project conception. Therefore, we acknowledge ARC Wessex staff contribution in the Acknowledgments section and their funding contribution in the Funding section/statement.

Please remove any funding-related text from the manuscript and let us know how you would like to update your Funding Statement. Currently, your Funding Statement reads as follows:

*“This study was funded by the National Institute for Health Research (NIHR) Applied Research Collaboration (ARC) Wessex. The views expressed in this publication are those of the author(s) and not necessarily those of the National Institute for Health and Care Research or the Department of Health and Social Care (https://www.arc-wx.nihr.ac.uk/).*

*The funders had no role in study design, data collection and analysis, decision to publish, or preparation of the manuscript.”*

Please include your amended statements within your cover letter; we will change the online submission form on your behalf.

We have removed the text “*previous NIHR-CLAHRC funded*” to read as “*previous NIHR project*”.

We are happy for the Funding Statement quoted above to be used in the manuscript.

3. Your ethics statement should only appear in the Methods section of your manuscript. If your ethics statement is written in any section besides the Methods, please move it to the Methods section and delete it from any other section. Please ensure that your ethics statement is included in your manuscript, as the ethics statement entered into the online submission form will not be published alongside your manuscript.

The ethics statement appears in the ‘Study design’ subsection of the Methods. At your request, we have deleted the ‘Ethics approval and consent to participate’ subsection of the Declarations, which previously read:

*“The current study was approved by the University of Southampton (ERGO ref: 60495.A2) and NHS HRA Research Ethics committees (IRAS ref: 288651).”*

**Review Comments to the Author**

**Reviewer #1:** This article reports the results of an interview study exploring the barriers and facilitators to the use of digital technology in supporting physical activity to manage LTC from the perspective of health care professionals and commissioners.

Generally, the article lacks clarity and would benefit from being more specific. For example, in parts the aim appears to be the use of digital technology to promote PA in the self-management of LTCs but in parts it appears the aim is to explore digital technology more generally.

We would like to thank and the reviewer for offering feedback and suggestions that allow us to improve the readability, coherence, and overall quality of our manuscript. Upon re-reading, we acknowledge areas which need more clarity and consistency in articulating our research. We have now revised the submission in line with your suggestions and recommendations.

In this ‘responses’ document, red text is used for our responses, with quotes from the revised manuscript italicised (blue-font in quotations highlighting text from the initial submission). Page and line numbers given here are in reference to the revised tracked changed manuscript, not the clean manuscript.

**Specific comments**

Abstract

1. The opening sentence appears incomplete.

Apologies for this oversight, we have inserted “*important*” to complete the opening sentence (page 2, line 18).

2. It would be helpful for non-UK audience to explain where Wessex is.

Wessex specifically encompasses Wiltshire, Dorset, Hampshire and Somerset. However, for brevity we have stated “*southern England*” in the Abstract (page 2, line 28). To note, the CRN Wessex region extends into other counties in southern England beyond Wessex. This is now also stated in the Methods, Sampling and Recruitment subsection (page 7, line 150).

3. State which population was purposively sampled and in which population convenience sampling was used.

General practitioners and healthcare professionals were recruited using purposive sampling, and commissioners via convenience sampling. We have revised this to now state:

*“Purposive sampling was used to recruit general practitioners and healthcare professionals, and convenience sampling to recruit commissioners (n=15).”*

Page 2, lines 29-30.

4. Make sure the conclusion aligns with the results (e.g., the conclusion talks about accessibility but this isn't mentioned in the results).

We have now aligned the Results and Discussion, including indication of which results/themes relate to the four NPT domains. We have also added the following sentence to the Abstract’s results:

*“In practice, this meant being technically, easy to use and culturally accessible (i.e., NPT: collective action – promoting healthcare work).”*	Page 2, lines 39-41.

We hope that the Conclusions now reads accurately and concisely, and preceded by ‘Strengths and limitations’ and ‘Implications for policy, practice and future research’, increases the paper’s potential for impact.

Introduction

5. The opening sentence talks about PA 'specifically to improve symptoms'. There needs to be a few sentences explaining how PA might improve symptoms.

Thank you for this suggestion. We have added the following sentence to describe how physical activity can potentially improve symptoms across long-term conditions. Page 4, lines 58-60.

*“Existing systematic reviews and guidelines make clear the benefits of physical activity in managing symptoms across LTCs, in addition to preventing complications and preserving function [2-6].”*

We also list some benefits of PA later in the Introduction (beginning of page 5, lines 87-89).

6. The sentence on line 92 appears incomplete.

Thank you. We have removed this sentence to highlight the relevance and importance of using the Normalisation Process Theory, whilst appraising the challenges of implementation particularly for digital tools in national health services.

Please refer to page 5, paragraph 3, lines 110-116.

7. How did the earlier scoping review inform this study?

To provide further explanation, we have revised page 6, lines 119-120 which now reads:

*“Our earlier scoping review [18]* *identified a range of digital tools to support the maintenance of physical activity for people with LTCs, their characteristics and their theoretical underpinnings.”*

Methods

8. Give examples of other healthcare professionals

We have now added *“… (e.g., nurses, physiotherapists and occupational therapists) …”* to indicate examples of healthcare professionals we initially intended to recruit.

Page 7, ‘Sampling and recruitment’, line 148.

9. Explain more about purposive sampling e.g., which characteristics were sampled and how people were identified and approached.

This is an excellent point. Cognisant of our word count, we have now provided this in-depth explanation in the Methods, instead we have added ‘*Solent NHS Trust*’ as a dissemination pathway, and the following:

*“**GPs and LTC healthcare specialists were sampled purposively from amongst Wessex CRN and Solent NHS Trust staff purposively to reflect diversity in geographical location, gender, age and LTC specialism/role. Wessex CRN and NHS Trust administrators emailed their clinical and primary care networks with information about the study. Potential participants then responded directly to staff in the CRN, NHS Trust or research teams to arrange an interview.”*

Page 7, ‘Sampling and recruitment’, lines 154-158.

10. Consider including the topic guide as supplementary material

Apologies for this oversight. The Interview topic guide has now been uploaded as S1Fig and referenced in the text (‘Data collection’, page 8, line 182).

11. Inclusion and exclusion criteria need to be clearly stated (e.g., did the interviewees have to have experience of promoting or using digital health in LTCs).

On revision we have added description of the inclusion criteria as:

*“Participants had to be NHS commissioners of services, GPs or healthcare professionals, have experience in providing care for people with LTCs, have recommended or used digital tools to support their LTC patients, be an English speaker and be aged ≥18 years.”*

Page 8, paragraph 2, lines 164-166

A broad definition of ‘digital tools’ was used in the recruitment materials, to allow various responses and gain insights into what our participant’s perceived as digital tools. To clarify this, we have added the following in the opening paragraph of the ‘Results’ (page 10, lines 223-224):

*“Digital tools were perceived by our healthcare professionals to range from apps to websites to Excel spreadsheets.”.*

Findings - this section would benefit from greater clarity with further development of the themes and ensuring that the themes relate to the use of digital tools in managing LTCs.

We have carefully reviewed our ‘Results’ section, and adjusted in response to the reviewer’s request. This includes modifying themes/sub-headings, and ensuring that the illustrative quotes used directly link to the NPT domains and themes. We have also clarified which barriers to use by healthcare professionals and commissioners, and which for LTC patients (including revising Table 2 to indicate barriers and facilitators). We are reporting, of course, perceptions of healthcare professionals on this matter. We present these amendments on pages 15-31.

This includes removal of the theme: "*Implementation of digital tools may pose a security risk*" and rewording each theme to read more succinctly. The issue of digital tools presenting a security risk was removed as too few participants raised this as a concern. However, we have kept mention of the issue under theme: “*Changing attitudes post-COVID-19*”, which was a facilitator, now placed under ‘cognitive participation’. See pages 25-26, lines 488-510.

In addition, we have revised the ‘Data analysis’ section to offer greater clarity on the development of the themes, and the theoretical constructs used, also responding to Reviewer 2. We explain that initial themes were developed, and then higher-level conceptual themes were developed by mapping to the constructs of the NPT framework. See page 9, lines 200-212.

12. Sentence on lines 187-188 lacks context/ justification.

We have removed this sentence for simplicity. We now briefly explain beneath Table 1 that:

*“Our findings are presented and interpreted in relation to the NPT [28], and focus on factors that healthcare professionals perceived to support successful implementation of digital interventions in healthcare (Table 2).”*

Page 15, lines 232-234.

13. Table 1 needs formatting, it is unclear which participant column 6 aligns. Consider splitting column 6 into two columns one stating which LTCs had been supported using digital technology and the other what digital technologies had been used.

We have reformatted the text in Tables 1 and 2 so that they are aligned in the top right corner of each cell. We have also added a single line space at the foot of each cell to increase spacing between text, and therefore clarity.

We have also divided column six as the reviewer suggests, which allows the reader to discern more clearly between: i) “*Types of LTCs supported*”, and ii) “*Digital tool(s) adopted*”. We hope that these changes enable readers to more easily interpret the table.

14. Why is asthma included in column 6 when no apps had been used?

We have removed all instances of “*Types of LTCs supported*”, where the participant did not use a digital tool. Please see Table 1 revision described above.

13. In LTCs listed in column 6 I suggest removing any conditions where digital technology hadn't been used to support the condition. As it stands the table is confusing as it appears that in column 6 in some cases all LTCs that the participant supports have been included and in other cases only LTCs that the participant has supported where digital technology has been utilised.

Please see the comment above, which we hope adds clarity and consistency.

14. Table 2 is confused and needs revising. It is unclear which domain aligns with which theme.

Please see our response above with the description of the revised layout for Table 2. We have also shortened the titles of each theme for clarity and readability.

We have restructured Table 2 to state each theme as either a barrier or facilitator; this includes making each theme heading more succinct. In the accompanying Results text, following Table 2, we now also indicate whether the theme is a barrier or facilitator (e.g., Barrier 1: condition-specific and accessible tools). We hope that this helps in interpretation and clarity.

15. Quotes need to be chosen which accurately reflect what the authors are trying to illustrate, at present the majority of the quotes don't do this. They are either not specific enough to illustrate the theme or they appear unrelated.

We have carefully reviewed our Results section and adjusted in response to the reviewer’s request. This includes modifying themes/sub-headings, and ensuring that the illustrative quotes used directly link to the NPT domains and themes. We have also clarified which themes were barriers and facilitators by our healthcare professionals and commissioners, including relevance to LTC patients (albeit based on professional interviews).

Please also refer to changes in Table 2.

16. The theme about tools being condition specific then contains a sentence about cultural specificity, this is a different theme to condition specificity and needs to be removed and placed in its own theme.

We agree with the reviewer’s suggestion that the theme: “*Digital tools should be condition-specific*” misleadingly contains a sentence and quote about cultural specificity. Our data contain too little reference to cultural specificity for it to represent a substantive theme in its own right.

We have instead moved it under the theme, “*Patient characteristics*” to which it relates better.

Page 27-29, lines 539-590.

17. What is meant by functionality. It is unclear what is meant by 'digital tools need to be functional' - is this the best word?

In our interviews, ‘*functionality’* or being ‘*functional’* referred to a tool/tools having usability (whether it was easy to use, would they use more regularly, were guidelines clear, was there too much information, and did they learn something new) and/or acceptability (whether it did what it claimed to do, was enjoyable to use, was accurate). On reflection, therefore, usability and acceptability may have been better terms than functionality or functional. We have, therefore, replaced ‘f*unctionality*’ / ‘*functional’* with ‘*usable and acceptable*’ throughout the manuscript.

We have also included the following for context in the Discussion.

*“**Technology that is well-designed from a usability perspective increases the utility of the technology/digital tool, reduce potential error, enhance user acceptance, and can consequently improve productivity [40].”*	Page 35, lines 742-744.

Including reference [40]: Ryan et al. 2015. *Health Systems, 4*:2, 151-8. <https://www.tandfonline.com/action/showCitFormats?doi=10.1057%2Fhs.2014.26>).

18. The theme digital tools involving voluntary sectors, is this referring to existing tools, potential tools or visiting website? This section would benefit from greater clarity.

Alongside explaining what our professional participants perceived as digital tools elsewhere in the manuscript, we have also revised the theme’s opening sentence and penultimate sentence, respectively, to clarify that ‘Voluntary sector involvement’ refers to:

*“…voluntary sector providers who either endorsed, developed, or provided access to digital tools…”*

Page 21, line 369-371.

*“Most healthcare professionals perceived endorsement, or development by, voluntary care providers, as an important factor …”*

Page 22, line 386-388.

We have also rephrased the theme to ‘*Voluntary sector involvement*’ for succinctness.

19. Investing time theme - who is investing the time? This theme would benefit from more explanation e.g. if the GP needs to invest time why do they need to? What is the purpose of investing the time?

The theme ‘*Investing time to use digital tools’* refers to the ‘professionals’ (i.e., commissioners and healthcare professionals) investing the time, but also indirectly, in investing time to appropriately support/educate their LTC patients whom they are recommending, supporting and/or monitoring using digital tools. We have now revised the theme title to: “*Professionals investing time in digital tools.*”

Please see Table 2 and theme ‘*Barrier 3: professionals investing time in digital tools’* (page 22, lines 391-409).

To clarify, on revision we have added following to conclude the theme:

*“Lack of time was a barrier for all healthcare professionals, in as far as they had no time to train, use, or teach patients to use the tools. Managers lacked time to deliver staff training and education. Participants also felt that the time demanded of patients in learning to use, and then using the tools for their own self-management was a major barrier.”*

Page 22, line 406-409.

20. It is unclear what Accurx has to do with digital tools and LTCs and self-management. This needs to be explained.

Thank you for raising this issue. We address the reviewer’s points through the revision to the section on the theme: “C*hanging attitudes post-COVID-19”*. This section now includes the following statement:

*“**Interestingly, the range of technologies that healthcare professionals and commissioners perceived as 'digital tools' facilitating self-management for people living with LTCs was wider than had originally been conceived when the study was designed and included technologies that were platforms rather than specific tools. For example, Accurx, a software provider offering an online platform that allowed them to communicate with their patients via SMS, email or the NHS app was valued by GPs and healthcare professionals.”*

Pages 25-26, line 492-497

21. How is the use of NHS mail related to digital tools for LTCs.

Please see above. NHS mail relates to our participant's wide conception of what 'digital tools' meant to them, specific to supporting the self-management of LTCs.

Discussion

22. This section would benefit from greater focus rather than re-iterating the results.

We are grateful of your suggestion, and in the revised Discussion section, we have i) reduced the descriptive content, and ii) incorporated new literature covering issues such as: healthcare professionals’ acceptance of digital tools (van der Ham et al. 2020), intervention development and testing for new digital tools, post-COVID-19 (Doyle et al. 2023), and professional networks influencing the implementation of digital tools (Brown et al. 2016).

Please see pages 32-38 for these revisions.

We have also revised the Limitations subsection (pages 38-39, lines 857-875, entitling it ‘Strengths and limitations’), introduced a new ‘Implications for policy, practice and future research’ subsection (pages 39-40, lines 877-898), and edited the Conclusions (page 40, lines 901-919).

23. Explain what is meant by 'digital divide'

On revision we have removed mention of digital divide, and instead focus on digital literacy, as the digital ‘divide’ did not come out of our interview data (whereas digital literacy was frequently mentioned).

24. Strengths and limitations could be expanded and better explained

We agree and have revised the ‘Strengths and limitations’ section. We have included discussion that our results are based on the experiences and perspectives of healthcare professionals and commissioners from a specific national region, and not the wider UK.

We have added a subheading to this section for structure and clarity (pages 38-39, lines 857-875).

25. Consider including a heading 'implications for policy and practice’.

We welcome and value your suggestion and have formed a new ‘*Implications for policy, practice and future research*’ subsection at the end of the Discussion.

Please see pages 39-40, lines 877-898.

Conclusions

26. Conclusions are not clearly linked to the results.

We agree with the reviewer’s comment and have revised the Conclusions section to i) align with the revised Abstract, Results and Conclusion, ii) removed focus on the NPT, and iii) streamlined content so that the word count affords an ‘*Implications for policy, practice and future research’* section. In rewriting we have aimed to ensure that our conclusions relate to directly to our original findings.

Please see page 40, lines 901-919.

**Reviewer #2:**

I wish to thank the editor for their invitation to review the manuscript. This is a qualitative study discusses the challenges of integrating physical activity into clinical practice for individuals with long-term conditions (LTCs), citing barriers like access, time constraints, and transport costs. Various digital tools are proposed as solutions to overcome these barriers and support widespread implementation to promote physical activity. Through semi-structured interviews conducted in Wessex, UK, with healthcare professionals and commissioners, the study explores needs, preferences, barriers, and facilitators to implementing digital tools for LTC self-management in NHS settings. Findings reveal that current digital tools lack specificity, functionality, and evidence-base, with suggestions for integration into health service IT systems, professional networks, and the importance of digital literacy for both professionals and patients. The study underscores the importance of evidence-based, endorsed digital tools that are integrated into existing health systems for successful LTC self-management.

Overall, I found the study could be potentially valuable to the field. However, there are a few areas that I think need improvement before it can be considered for publication.

1.	The manuscript needs to be heavily edited for clarity. Clear and concise writing is essential to ensure that readers can understand the research findings and their implications. For example, throughout the manuscript there is a lack of differentiation between barriers to HCPs and barriers to patients. These should be separated, to provide clarity on the distinct challenges faced by each group. Separating the barriers would enable a more focused analysis of the unique factors influencing the adoption and implementation of digital tools from both perspectives.

We thank you for your constructive advice and we have now addresses issues with clarity and concision, in response to reviewer 1’s points, in: i) ensuring that the Results align to the Discussion and Conclusion, ii) revising Table 2 to better illustrate the themes in relation to NPT domains, and iii) replacing quotations to better represent/align to NPT domains and themes. Please see our responses to reviewer 1 above.

2.	There is a lack of clarity in conceptual framework: While the manuscript mentions the use of the Consolidated Framework for Implementation Research (CFIR) and the Normalization Process Theory (NPT), it fails to provide clear explanations regarding which domains of the CFIR framework were probed during the interviews. Similarly, the Normalization Process Theory is briefly mentioned, but its application and relevance to the study are not adequately explained. It is crucial for readers to understand how these theoretical frameworks were operationalized and utilized in the study to assess barriers and facilitators to the implementation of digital tools in healthcare settings.

We are grateful to the reviewer for this observation and have carefully reviewed our Data Collection and Data Analysis sections, with regard to the use of the CFIR and NPT conceptual frameworks.

For clarification, the CFIR informed the development of the interview guide, but was not the focus of interview data collection, nor data analysis and the development of themes. On revision we have removed CFIR from Fig 1 footnote and the manuscript Results. We now only mention CFIR in the Data collection subsection, regarding it’s use to inform the interview guide development.

Please refer to page 8, lines 185-191.

In response to the reviewer’s comment about the NPT, we now introduce and define NPT and explain its application and relevance. We drew on NPT to help explain and understand the themes developed from our qualitative study. NPT enabled us to understand the factors shaping the implementation of digital tools in practice, from the perspectives of healthcare professionals and commissioners.

Please see the Introduction (page 5, paragraph 3, lines 109-118), where we explain what the NPT is, how it can be applied, it’s constructs/domains, and why we adopted it for this particular study. Also, the Data analysis (page 9, lines 207-211), where we explain how the interview themes were mapped to the NPT domains, and Results, whereby we present the interview themes assigned to the NPT’s domains (Table 2, pages 15-16, lines 239-241).

3. **Discussion of Findings:** The discussion of findings in the manuscript lacks depth. There is a need for a more thorough analysis and interpretation of the interview data, particularly in relation to the identified barriers and facilitators to the implementation of digital tools in healthcare settings. Additionally, the manuscript could benefit from a more robust comparison of findings with existing literature.

We agree with the reviewer’s comment. Please see our response to Reviewer 1 above for revisions, including specific revisions:

- ‘Strengths and limitations’ - pages 38-39, lines 857-875
- ‘Implications for policy, practice and future research’ - pages 39-40, lines 877-898
- ‘Conclusions’ -page 40, lines 901-919.

In summary, while the manuscript addresses an important topic, its current presentation lacks clarity and depth in several key areas. I believe that significant revisions are necessary to strengthen the manuscript and ensure its suitability for publication in PLOS ONE.

We hope that the revisions made in response to reviewer’s comments have improved the quality and clarity of our manuscript. We are grateful for the detailed consideration of our manuscript by reviewer’s and for the opportunity to address their suggestions.

Thank you once again for your time and consideration.

Kindest regards,

Dr James Gavin (on behalf of all co-authors)

Lecturer in musculoskeletal health

E-mail: [J.P.Gavin@soton.ac.uk](mailto:J.P.Gavin@soton.ac.uk)
